# A multicenter, real‐world analysis of primary central nervous system lymphoma in those with and without human immunodeficiency virus

**DOI:** 10.1002/jha2.474

**Published:** 2022-06-02

**Authors:** Christopher Dittus, Natalie Grover, Tarsheen Sethi, Jonathon B. Cohen, Alfredo Voloschin, Janhvi Rabadey, Xianming Tan, Anne Beaven, Steven I. Park

**Affiliations:** ^1^ Division of Hematology University of North Carolina at Chapel Hill Chapel Hill North Carolina USA; ^2^ Division of Hematology Yale University New Haven Connecticut USA; ^3^ Department of Hematology and Medical Oncology Emory University‐Winship Cancer Institute Atlanta Georgia USA; ^4^ Department of Biostatistics University of North Carolina at Chapel Hill Chapel Hill North Carolina USA; ^5^ Atrium Health Levine Cancer Institute Charlotte North Carolina USA

**Keywords:** central nervous system lymphoma, chemotherapy, HIV, non‐Hodgkin lymphoma, outcomes

## 1 INTRODUCTION

Primary central nervous system lymphoma (PCNSL) is a type of extranodal non‐Hodgkin lymphoma (NHL) involving the brain, leptomeninges, eyes, spinal cord, and/or cerebrospinal fluid (CSF) [1]. Importantly, a diagnosis of PCNSL can only be made if there is no evidence of systemic NHL. PCNSL is a rare disease with a yearly incidence of 0.3 cases per 100,000 [2].

Despite improvements in treatment over the past two decades, many patients still have poor outcomes. Patients with human immunodeficiency virus (HIV) PCNSL have been shown to have particularly poor outcomes, although the reason for this is not well understood [3]. Most clinical trials in PCNSL exclude this population and retrospective studies assessing the management of HIV PCNSL are scarce.

In our study, we aimed to report the real‐world survival of PCNSL at three large academic US centers, including those who received high‐dose methotrexate (HD‐MTX) versus those who did not. We also compared the survival of HIV PCNSL relative to non‐HIV PCNSL and evaluated factors that may account for differences in clinical outcomes among various subgroups.

## 2 METHODS

The medical records were queried for patients ≥ 18 years of age newly diagnosed with PCNSL at 3 academic US medical centers (University of North Carolina Medical Center, Vanderbilt University Medical Center, Emory University Hospital). The dates of diagnosis occurred between January 2004 and July 2020. Inclusion criteria included only the diffuse large B‐cell lymphoma (DLBCL) histology. Cases were excluded if there was any disease outside of the central nervous system (CNS) or if they previously had systemic DLBCL. Demographic information was collected from the medical record, as well as disease‐related information, HIV status, HD‐MTX treatment (defined as ≥ 3 g/m^2^) with or without other chemotherapeutic agents, imaging to determine progression, and survival data. Data were analyzed for the entire cohort and separately for HIV and non‐HIV groups. Demographic variables were summarized using appropriate statistics (frequencies, mean and standard deviation) and compared between HIV and non‐HIV patients using Fisher's exact tests or Wilcoxon rank‐sum tests. Progression‐free survival (PFS) and overall survival (OS) rates were compared between HIV and non‐HIV patients using log‐rank tests. We also conducted multivariate analyses on PFS and OS using Cox proportional hazard regression model. This study was approved by the Institutional Review Board of each participating site and is in accordance with the Declaration of Helsinki.

## 3 RESULTS

### 3.1 PCNSL cohort

We identified 158 cases of PCNSL (Table 1). The median age for the entire cohort was 59 and 55% identified as male. We evaluated for high‐risk features including performance status (PS) ≥ 2 (32%), deep structure involvement (53.8%), positive CSF protein (60.2%), and elevated lactate dehydrogenase (LDH) (42.9%). Additionally, 79.1% of patients received frontline HD‐MTX and 8.9% received frontline radiation therapy (not including consolidation). Frontline chemotherapy regimens were: HD‐MTX alone (43.2%), MPV (methotrexate, procarbazine, and vincristine; 34.4%), MT (methotrexate and temozolomide; 8.8%), HD‐MTX/cytarabine (7.2%), and other regimens (6.4%). Nine of 151 evaluable patients received autologous stem cell transplant consolidation after frontline therapy

**TABLE 1:** Patient characteristics of a PCNSL cohort by HIV status

	Total	HIV	Non‐HIV	*p*‐value*
Age (median/range)	59 (18–84)	40 (20–59)	61 (18–84)	<0.0001*
Gender	Male: 87 (55%) Female: 71 (45%)	Male: 17 (65%) Female: 9 (35%)	Male: 70 (53%) Female: 62 (47%)	0.30
PS ≥ 2	49/153 (32%)	15/26 (57.7%)	34/127 (26.8%)	0.005*
Deep Structure^**^	85/158 (53.8%)	16/26 (59.3%)	69/132 (52.6%)	0.50
Elevated CSF Protein	65/108 (60.2%)	17/24 (70.8%)	48/84 (57.1%)	0.20
LDH elevated	60/140 (42.9%)	13/25 (52%)	47/115 (40.9%)	0.40
Frontline MTX	125/158 (79.1%)	14/26 (53.8%)	111/132 (84%)	0.001*
Frontline XRT^#^	14/158 (8.9%)	5/26 (19.2%)	9/132 (6.8%)	0.06

^*^ Statistically significant by Fisher's exact test or Wilcoxon rank‐sum test; HIV versus non‐HIV.

^**^ Periventricular, basal ganglia, brainstem, cerebellum.

^#^ Consolidation XRT was excluded.

PCNSL: Primary central nervous system lymphoma; HIV: human immundeficiency virus; PS: Performance status; CSF: cerebrospinal fluid; MTX: methotrexate, XRT: radiation therapy.

One hundred fifty‐seven of 158 cases were evaluable for survival. The median PFS for the entire cohort was 1.18 years and the median OS was 3.24 years. Patients who received HD‐MTX had a significant improvement in PFS (1.69 years vs. 0.25 years; *p *= 0.0014) and OS (4.01 years vs. 1.05 years; *p *= 0.00067) compared to those who did not receive HD‐MTX.

### 3.2 HIV versus non‐HIV PCNSL

Twenty‐six of the 157 cases were HIV positive (Table 1). Patients with HIV PCNSL were significantly younger than those with non‐HIV PCNSL (40 years vs. 61 years, *p *= < 0.0001) and had a worse performance status (PS ≥ 2: 57.7% vs. 26.8%, *p *= 0.005). Notably, patients with HIV were less likely to receive HD‐MTX compared to patients without HIV (53.8% vs. 84%, *p *= 0.001). Patients who received HD‐MTX were also older (58 years vs. 51 years, *p *< 0.05) and had an improved PS (ECOG 1.1 vs. 1.8, *p *< 0.05). The frequency of receiving frontline whole‐brain radiation therapy (WBRT) trended towards significance in favor of the HIV PCNSL group. Other factors such as gender, deep structure involvement, elevated CSF protein, and elevated LDH were not significantly different between the groups.

**FIGURE 1:**
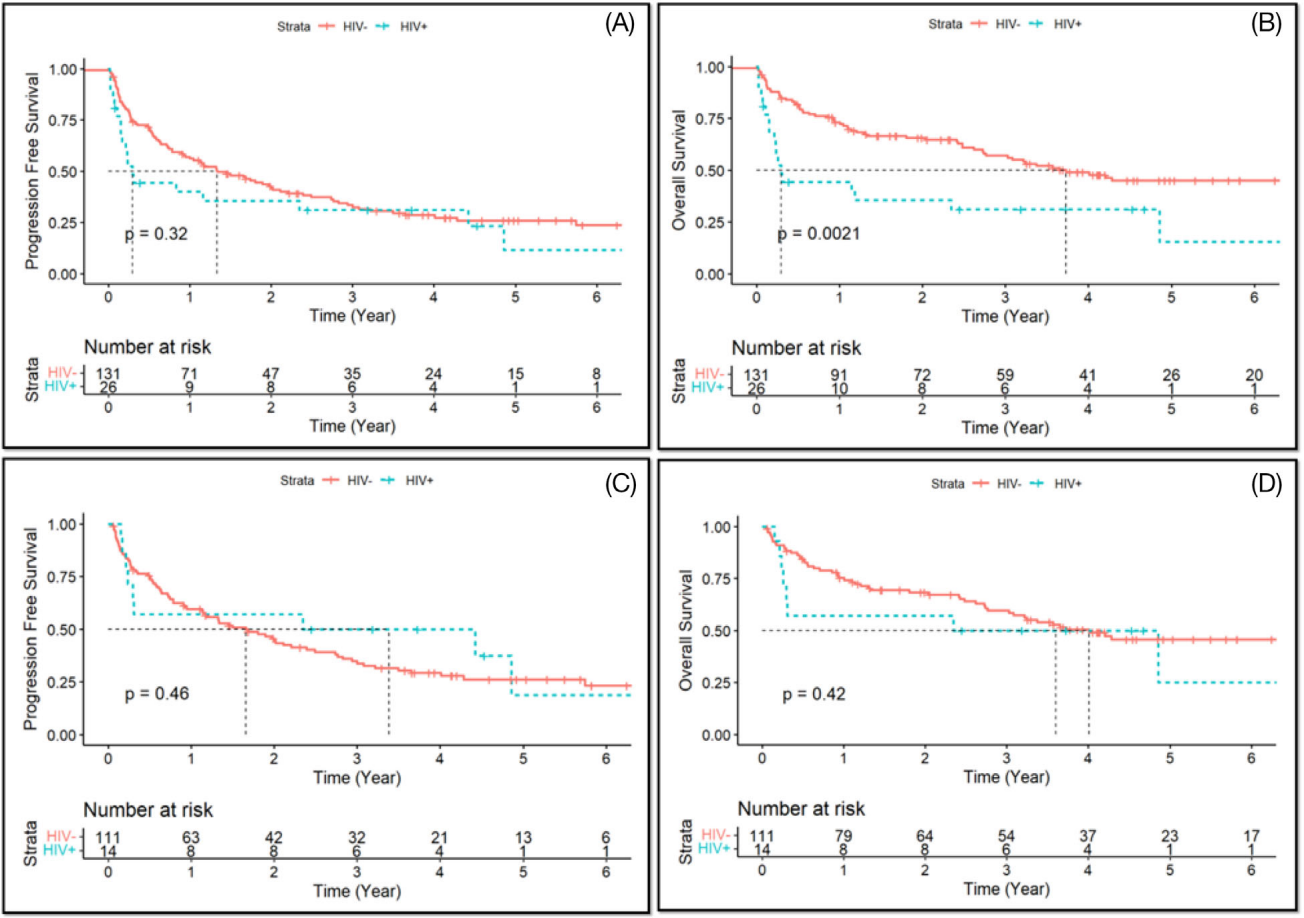
HIV PCNSL versus non‐HIV PCNSL (whole cohort): Progression free survival (A) and overall survival (B). HIV PNCSL versus non‐HIV PCNSL (HD‐MTX): Progression free survival (C) and overall survival (D). HIV: Human immunodeficiency virus; PCNSL: Primary central nervous system lymphoma; HD‐MTX: high‐dose methotrexate

When HIV PCNSL was compared to non‐HIV PCNSL, there was not a statistically significant difference in PFS (0.30 years vs. 1.34 years; *p *= 0.32), but there was a statistically significant difference in OS (0.30 years vs. 3.73 years; *p *= 0.0021) (Figure 1). When comparing only those patients who received HD‐MTX in the HIV group vs. non‐HIV group, there was not a statistically significant difference in PFS (3.39 years vs. 1.66 years; *p *= 0.46) or OS (3.6 years vs. 4.0 years; *p *= 0.42) (Figure 1).

### 3.3 Multivariate analysis

In a multivariate analysis (MVA), we evaluated the impact of each of the following variables on PFS and OS: PS, age, HIV status, and treatment with HD‐MTX. Increasing age correlated with a higher risk of progression (HR = 1.02, *p *= 0.01; 2% risk/year), and treatment with HD‐MTX resulted in a decreased risk of progression (HR = 0.50, *p *= 0.003). PS and HIV status did not have a significant impact on PFS. In terms of OS, each of the following factors had a statistically significant impact: increasing age (HR = 1.03, *p *= 0.001; 3% risk/year), treatment with HD‐MTX (HR = 0. 46, *p *= 0.003), and HIV status (HR = 3.16, *p *= 0.001). PS did not have a significant impact on OS.

### 3.4 HIV subset analysis

In a subset of HIV PCNSL patients at the University of North Carolina (*N* = 19), 63.2% of patients had a CD4 count <50 at diagnosis of PCNSL. Additionally, 42% had newly diagnosed HIV at the time of PCNSL presentation, while only 36.8% had been on ART for ≥3 months prior to PCNSL diagnosis. These variables were also compared according to HD‐MTX status. Those who received HD‐MTX (*N* = 10) did not have a statistically significant difference in any HIV‐related factors compared to those who did not receive HD‐MTX (*N* = 9).

## 4 DISCUSSION

In one of the largest real‐world analyses of PCNSL to date, we found a median PFS of 1.18 years and a median OS of 3.24 years in patients with previously untreated PCNSL, including HIV PCNSL. As expected with real‐world data, survival rates were lower compared to a prospective trial evaluating a HD‐MTX induction regimen followed by WBRT consolidation, which had a median PFS of 3.3 years and median OS of 6.6 years [4]. Studies evaluating a HD‐MTX induction regimen with autologous stem cell transplant consolidation have shown 2‐year survival rates of approximately 80% [5, 6]. In each of these studies, the median age (57‐60 years) was similar to our cohort (59 years). Our study had nearly a third of patients with a PS ≥ 2, included patients with HIV, and included those who did not receive HD‐MTX. These patient factors are important to consider when reviewing real‐world data.

To evaluate this further, we compared patients who were able to receive treatment with HD‐MTX with those who were not. As expected, those who received HD‐MTX had significantly improved PFS and OS. This was further supported by the MVA, which showed that treatment with HD‐MTX was associated with improved PFS and OS independent of age, PS or HIV status. This lends further support to HD‐MTX‐based regimens as the standard frontline treatment for PCNSL, and most patients should be offered HD‐MTX‐based induction. However, there is likely selection bias in this non‐randomized study that could contribute to these findings. Not all patients are candidates for HD‐MTX due to severe renal failure or other comorbid conditions, and they may have worse survival due to these factors. Further research is needed to identify alternatives for these patients.

HIV PCNSL has been evaluated in several retrospective, and few prospective, studies [3, 7–10]. In our analysis, only 16.6% of PCNSL cases were HIV positive. We found that HIV PCNSL had a significantly worse OS compared to non‐HIV PCNSL. Interestingly, the difference in PFS between these groups was not significantly different. In the HIV PCNSL group, the median PFS and OS were both 0.3 years, while the PFS was 1.34 years and the OS was 3.73 years in the non‐HIV PCNSL group. Of the patients with a documented cause of death, the majority were from PCNSL. This suggests that HIV PCNSL does poorly in the frontline setting and has virtually no effective salvage options. Alternatively, non‐HIV PCNSL appears to have more options at relapse, which could account for the improved OS relative to HIV PCNSL.

In order to further evaluate the reasons for the discrepancy between HIV and non‐HIV PCNSL survival, we compared only those patients who received frontline HD‐MTX. This revealed that in those receiving HD‐MTX, the discrepancy in OS between HIV and non‐HIV PCNSL was no longer statistically significant. This was likely driven by the improvement in OS from 0.3 years to 3.6 years in the HIV PCNSL patients who received HD‐MTX. Therefore, it seems that at least part of the difference in survival is being driven by the lower use of HD‐MTX in certain HIV PCNSL patients. When we compared patient and disease factors between HIV and non‐HIV PCNSL patients, we found that a PS ≥ 2 was significantly more common in the HIV PCNSL group. This could account for some of the reason why these patients did not receive HD‐MTX. The HIV PCNSL cohort was significantly younger than the non‐HIV PCNSL group, so older age was not a factor in this discrepancy. Despite the potential impact of these contributing factors, the MVA revealed that HIV status is associated with worse OS independent of HD‐MTX, age, or PS. Age and HD‐MTX were also independently associated with worse OS. The fact that HIV PCNSL patients had worse PS and received HD‐MTX at lower rates likely compounded the abysmal survival in this group.

## 5 CONCLUSION

In this large retrospective analysis of PCNSL, we have shown that real‐world survival is poor compared to clinical trial results. Additionally, HIV PCNSL has worse OS than non‐HIV PCNSL. This difference was not seen when evaluating only patients who have received HD‐MTX. HIV PCNSL patients received HD‐MTX at lower rates, likely due to worse baseline PS. The MVA showed that HIV status, age, and receiving HD‐MTX all independently contributed to survival outcomes, with HIV status having the largest magnitude effect. Every patient with PCNSL should be considered for treatment with HD‐MTX regardless of HIV status, and new treatment approaches for relapsed HIV PCNSL need to be evaluated.

## CONFLICT OF INTEREST

Christopher Dittus: Seagen, BeiGene and Genmab. Natalie Grover: Genentech, ADC Therapeutics, Kite, Novartis, Tessa Therapeutics. Jonathon B. Cohen: BMS, Genentech, LOXO, BioInvent, Novartis, Takeda, Astra Zeneca, Loxo, Beigene, Kite, Pharmacyclics, Abbvie. Steven I. Park: BMS, Seagen, Morphosys, Epizyme. Tarsheen Sethi, Alfredo Voloschin, JR, Xianming Tan, and Anne Beaven: No conflict of interest to disclose.

## ETHICS STATEMENT

This study is in accordance with the Declaration of Helsinki for the ethical treatment of human research participants.

## FUNDING INFORMATION

The authors received no specific funding for this work.

## Data Availability

Data can be provided upon request.
